# Supplementary material for: Semi-quantitative detection of pseudouridine modifications and type I/II hypermodifications in human mRNAs using direct long-read sequencing
Source: Nat Commun. 2023 Jan 19;14:334. doi: 10.1038/s41467-023-35858-w (PMC9852470; doi:10.1038/s41467-023-35858-w)
Supplement: Supplementary file 2 — Description of Additional Supplementary Files [file 41467_2023_35858_MOESM2_ESM.pdf]

Description of Additional Supplementary Files

File Name: Supplementary Data 1
Description: Candidate psi sites in ribosomal RNA

File Name: Supplementary Data 2
Description: Validated psi site in mature mRNA sequences

File Name: Supplementary Data 3
Description: Psi sites detected by direct RNA sequencing

File Name: Supplementary Data 4
Description: Hypermodification type I psi sites

File Name: Supplementary Data 5
Description: Hypermodification type II psi sites

File Name: Supplementary Data 6
Description: GO analysis of psi sites

File Name: Supplementary Data 7
Description: Oligonucleotide sequences used for synthetic controls and sequencing primers
