# Supplementary material for: Semi-quantitative detection of pseudouridine modifications and type I/II hypermodifications in human mRNAs using direct long-read sequencing
Source: Nat Commun. 2023 Jan 19;14:334. doi: 10.1038/s41467-023-35858-w (PMC9852470; doi:10.1038/s41467-023-35858-w)
Supplement: Supplementary file 10 — Reporting Summary [file 41467_2023_35858_MOESM10_ESM.pdf]

nature portfolio

Corresponding author(s): Sara H. Rouhanifard

Last updated by author(s): 11/10/2022

Reporting Summary

Nature Portfolio wishes to improve the reproducibility of the work that we publish. This form provides structure for consistency and transparency in reporting. For further information on Nature Portfolio policies, see our Editorial Policies and the Editorial Policy Checklist.

Statistics

For all statistical analyses, confirm that the following items are present in the figure legend, table legend, main text, or Methods section.

n/a	Confirmed	
☐	☒	The exact sample size (*n*) for each experimental group/condition, given as a discrete number and unit of measurement
☐	☒	A statement on whether measurements were taken from distinct samples or whether the same sample was measured repeatedly
☐	☒	The statistical test(s) used AND whether they are one- or two-sided<br>*Only common tests should be described solely by name; describe more complex techniques in the Methods section.*
☐	☒	A description of all covariates tested
☐	☒	A description of any assumptions or corrections, such as tests of normality and adjustment for multiple comparisons
☐	☒	A full description of the statistical parameters including central tendency (e.g. means) or other basic estimates (e.g. regression coefficient) AND variation (e.g. standard deviation) or associated estimates of uncertainty (e.g. confidence intervals)
☐	☒	For null hypothesis testing, the test statistic (e.g. *F*, *t*, *r*) with confidence intervals, effect sizes, degrees of freedom and *P* value noted<br>*Give P values as exact values whenever suitable.*
☒	☐	For Bayesian analysis, information on the choice of priors and Markov chain Monte Carlo settings
☐	☒	For hierarchical and complex designs, identification of the appropriate level for tests and full reporting of outcomes
☒	☐	Estimates of effect sizes (e.g. Cohen's *d*, Pearson's *r*), indicating how they were calculated

Our web collection on statistics for biologists contains articles on many of the points above.

Software and code

Policy information about availability of computer code

Data collection	guppy (3.2.10), minimap 2 (2.17), samtools (2.8.13), nanopolish (0.13.2), enrichR (2016 update), IGV (2.8.13), MinKnow (19.12.5)
Data analysis	Scripts for all analyses presented in this paper, including all data extraction, processing, and graphing steps are freely accessible at https://github.com/RouhanifardLab/PsiNanopore.git.

For manuscripts utilizing custom algorithms or software that are central to the research but not yet described in published literature, software must be made available to editors and reviewers. We strongly encourage code deposition in a community repository (e.g. GitHub). See the Nature Portfolio guidelines for submitting code & software for further information.

Data

Policy information about availability of data

All manuscripts must include a data availability statement. This statement should provide the following information, where applicable:

- Accession codes, unique identifiers, or web links for publicly available datasets
- A description of any restrictions on data availability
- For clinical datasets or third party data, please ensure that the statement adheres to our policy

Data availability
All code used in this work is publicly available at github.com/RouhanifardLab/PsiNanopore. All FASTQ files and Fast5 raw data generated in this work have been made publicly available in NIH NCBI SRA under the BioProject accession PRJNA777450. All other data are available from the authors upon reasonable request.

Field-specific reporting

Please select the one below that is the best fit for your research. If you are not sure, read the appropriate sections before making your selection.

☒ Life sciences ☐ Behavioural & social sciences ☐ Ecological, evolutionary & environmental sciences

For a reference copy of the document with all sections, see nature.com/documents/nr-reporting-summary-flat.pdf

Life sciences study design

All studies must disclose on these points even when the disclosure is negative.

Sample size	We observed that the base quality on sites that have 3 or fewer reads from a single run is low, relative to the rest of the population, which would create bias in the downstream analysis (Fig. 2a). Therefore, to ensure sufficient coverage in both the direct RNA and IVT samples, we set a minimum threshold of 7 reads represented two or more biological replicate before evaluation for site modification. Three independent biological replicates were used on three different flow cells and compared to two independent biological replicates of unmodified transcriptome controls performs on two different flow cells.
Data exclusions	No data were excluded from the analysis
Replication	Identified sites were called based on their probability score in both biological replicates. Identified sites were benchmarked and verified against previously existing data sets.
Randomization	Randomization is not relevant to this study because direct RNA samples were compared to unmodified transcriptomes.
Blinding	Blinding was not relevant to this study because we were comparing the signals obtained from direct RNA sequencing to an unmodified transcriptome negative control.

Reporting for specific materials, systems and methods

We require information from authors about some types of materials, experimental systems and methods used in many studies. Here, indicate whether each material, system or method listed is relevant to your study. If you are not sure if a list item applies to your research, read the appropriate section before selecting a response.

Materials & experimental systems

n/a	Involved in the study	
☒	☐	Antibodies
☐	☒	Eukaryotic cell lines
☒	☐	Palaeontology and archaeology
☒	☐	Animals and other organisms
☒	☐	Human research participants
☒	☐	Clinical data
☒	☐	Dual use research of concern

Methods

n/a	Involved in the study	
☒	☐	ChIP-seq
☒	☐	Flow cytometry
☒	☐	MRI-based neuroimaging

Eukaryotic cell lines

Policy information about cell lines

Cell line source(s)	HeLa cells, ATCC
Authentication	ATCC uses morphology, karyotyping, and PCR-based approaches to confirm the identity of human cell lines.
Mycoplasma contamination	Cell lines tested negative for mycoplasma.
Commonly misidentified lines (See ICLAC register)	no commonly misidentified lines were used in this study
